# Efficacy and Safety of Ab Externo Open Conjunctiva XEN^®^ 63 µm Implantation with a 30G Needle Scleral Tract in Primary Open-Angle Glaucoma

**DOI:** 10.3390/jcm14093195

**Published:** 2025-05-05

**Authors:** Yann Bertolani, Jaume Rigo-Quera, Laura Sánchez-Vela, Olivia Pujol-Carreras, Manuel Amilburu, Antonio Dou, Marta Castany

**Affiliations:** Ophthalmology, Vall d’Hebron University Hospital, 08035 Barcelona, Spain; jaume.rigo@vallhebron.cat (J.R.-Q.); laura.sanchezvela@vallhebron.cat (L.S.-V.); olivia.pujol@vallhebron.cat (O.P.-C.); manuel.amilburu@vallhebron.cat (M.A.); antonio.dou@vallhebron.cat (A.D.); marta.castany@vallhebron.cat (M.C.)

**Keywords:** primary open-angle glaucoma, XEN63 µm, minimally invasive glaucoma surgery, intraocular surgery, open conjunctiva approach, survival analysis

## Abstract

**Background**: This study aimed to assess the efficacy and safety of the 30G needle mediated ab externo open conjunctiva approach for the XEN 63 µm implant in primary open-angle glaucoma. **Methods**: A retrospective and non-randomized study was conducted on consecutive cases of medically refractory primary open-angle glaucoma treated with standalone ab externo open conjunctiva XEN^®^ 63 µm (North Chicago, Illinois) with one-year follow-up. **Results**: Twenty-two eyes were included. The mean preoperative intraocular pressure was 21.9 ± 7.2 mmHg, and the mean number of glaucoma medications was 2.4 ± 0.9. All patients underwent mitomycin 0.02% application for 2 min, and Healaflow^®^ (MedicalMix, Spain), was implanted in 11 cases (50%). Complete surgical success was achieved in 14 cases (63.6%). No statistical differences in complete surgical success were noted based on the use of Healaflow^®^. A significant reduction in intraocular pressure (11.8 ± 3.4 mmHg) and in the number of hypotensive medications (0.2 ± 0.5 mmHg) was observed 1 year after the procedure. Transient hypotony was detected in 31.8% of cases. Complications secondary to hypotony included four cases of serous choroidal detachment and one case of localized hemorrhagic choroidal detachment, the latter associated with hypotonic keratopathy and hypotonic maculopathy. All these complications evolved favorably with conservative management and adjusted topical treatment. **Conclusions:** This study highlights the efficacy and safety of this approach for the XEN 63 µm implant in medically refractory primary open-angle glaucoma.

## 1. Introduction

Primary open-angle glaucoma (POAG) is one of the leading causes of irreversible blindness worldwide, characterized by progressive optic neuropathy and associated visual field loss. Reducing intraocular pressure (IOP) is the major modifiable factor to slow the progression of the disease, and may be achieved through pharmacological treatment, laser, or surgery. Trabeculectomy (TBT) and non-penetrating deep sclerectomy (NPDS) have been the standard treatment for medically refractory POAG [1,2]. However, these surgeries may convey sight-threatening complications, especially related to hypotony, such as choroidal detachment and hypotonic maculopathy. Hence, highly myopic and aphakic patients are particularly prone to developing these adverse effects along the postoperative course [3,4]. Therefore, to minimize these potential risks, minimally invasive glaucoma surgery (MIGS) has been developed, representing a safer approach [5,6]. MIGS devices may be classified according to their drainage route into trabecular (iStent, Hydrus), suprachoroidal (iStent Supra), subconjunctival devices (XEN, PreserFlo) and trabecular meshwork ablative techniques (GATT, Trabectome). The XEN^®^ Gel Stent implant (North Chicago, IL, USA) decreases IOP by facilitating the drainage of aqueous humor from the anterior chamber to the subconjunctival space. It is a non-valved device based on the Hagen–Poiseuille principle, designed to reduce the incidence of early postoperative hypotony and associated complications [7]. As its mechanism depends on the formation of a filtration bleb, some authors have advocated classifying the XEN implant as a minimally invasive bleb surgery (MIBS) device [7,8]. 

Initially, the XEN^®^ Gel Stent implant was designed to be implanted with an ab interno with closed conjunctiva (AIC) approach. However, in recent years, the off-label ab externo approach (both with closed and open conjunctiva) has been described, gaining some popularity. Currently, two models are approved for use in the European Union and the United States, based on the lumen diameter: XEN^®^ 45 µm and XEN^®^ 63 µm. Pirani et al. [9], Yuan et al. [10], and El Helwe et al. [11] evaluated and evidenced the efficacy and safety of the ab externo approach for the XEN^®^ 45 µm device in POAG. Moreover, its efficacy has been reported for steroid-induced glaucoma and refractory glaucoma with prior failed tube by Tan et al. [12] and To et al. [13], respectively. Although the efficacy and safety of the AIC XEN^®^ 63 µm has been reported by Martinez-de-la-Casa et al. [14], Fea et al. [15], and Lavin-Dapena C et al. [16], no studies have been conducted concerning the ab externo with open conjunctiva (AEO) XEN^®^ 63 µm. This is the first study to address both the efficacy and safety of the AEO approach for the XEN^®^ 63 µm in POAG and to describe the use of a 30G needle to perform the scleral tract.

## 2. Materials and Methods

### 2.1. Study Design and Ethics Statement 

This is a retrospective, non-randomized, single-center, and uncontrolled study of consecutive patients with medically uncontrolled POAG who underwent standalone AEO XEN 63 µm, with a minimum follow-up of one year at the Glaucoma Department of the Vall d’Hebron University Hospital. The approval from our Institutional Review Board (CEIm of the Vall Hebron University Hospital) was obtained for the review of the patient’s clinical records (Protocol PS(AG)019/2024(6350), November 2024). This research complies with the Good Clinical Practice/International Council for Harmonization Guidelines and adheres to the Declaration of Helsinki.

### 2.2. Study Participants and Data Collection 

This study included consecutive patients with medically uncontrolled POAG who underwent a standalone AEO XEN 63 µm implant with evaluation at day 1; week 1; month 1, 2, 3, and 6; and 1 year after the procedure. The inclusion criteria were age over 18 years, diagnosis of POAG, Shaffer angle ≥ 3, ability to provide informed consent, and a minimum follow-up of 1 year. If the surgery was performed bilaterally, both eyes were included in the study. Patients with any form of glaucoma other than POAG, any surgical procedure (including cataract surgery) 6 months prior to surgery, pregnancy, vitreous in the anterior chamber, presence of intraocular silicone oil, or allergy to any medication required for implantation or any of the device components were excluded. Also, patients with previous glaucoma surgery were excluded from this study. Patients underwent surgery in the following scenarios: unmet target IOP despite maximum tolerated topical medical therapy, intolerance to medical therapy, and documented structural glaucomatous progression, with or without accompanying functional deterioration.

The electronic medical records were accessed to collect the data. Recorded baseline characteristics included age, sex, laterality, preoperative visual field acuity (logMAR), preoperative IOP using Goldmann Applanation Tonometry, number of glaucoma medications, previous selective laser trabeculoplasty (SLT), previous glaucoma surgeries, preoperative lens status, and mean deviation (MD) in SITA fast 24-2 program visual field (VF) (Carl Zeiss Meditec, Inc., Oberkochen, Germany). All patients underwent a thorough ophthalmological examination, including a review of the medical and ophthalmological history, IOP measurements, gonioscopy, and glaucoma staging through optic nerve head optical coherence tomography (ONH-OCT) (Cirrus^®^ HD-OCT 500, Zeiss, Germany) and 24.2 VF (Humphrey Field Analyzer 3^®^, Zeiss, Germany).

Postoperative visit regime (day 1; week 1; month 1, 2, 3, and 6; and annually) included ophthalmic examination with IOP measurements. The requirements in needlings, ocular hypotensive medication, and other surgical procedures were recorded. Anterior segment optical coherence tomography (AS-OCT) (Anterion^®^, Heidelberg, Germany) to assess the bleb morphology and filtration was performed at the ophthalmologist’s discretion. ONH-OCT and 24.2º VF were performed to assess functional or structural progression at 6 months and 1 year after the surgery. Intraoperative and postoperative adverse events, the need for surgical bleb revision, and additional required glaucoma surgeries were recorded. The surgeries were performed by different ophthalmologists with expertise in glaucoma surgery.

### 2.3. Surgical Technique 

**Figure 1:**
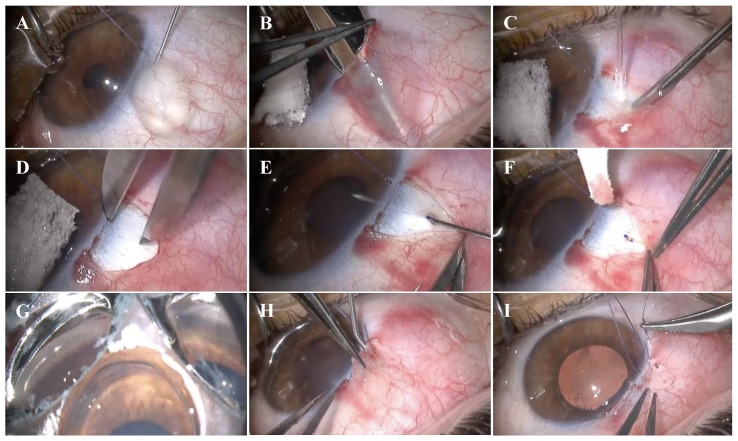
Surgical technique for 30-G needle-mediated ab externo XEN^®^ 63 µm implant. (**A**) Instillation of sub-Tenon’s anesthetic (2% lidocaine with epinephrine) after initial opening with Vanna’s scissors. (**B**) Superior fornix-based conjunctival flap with dissection of conjunctiva and Tenon’s capsule. (**C**) Application of diathermy on the scleral bed. (**D**) Marking at 2.0 mm from the surgical limbus. (**E**) Scleral tract performed with a 30G needle. (**F**) Manual insertion of the XEN® 63 implant using non-toothed forceps. (**G**) Verification of correct device implantation in the anterior chamber via gonioscopy lens. (**H**) Checking sub-Tenon’s XEN colocation and placement of sub-Tenon’s Healaflow^®^. (**I**) Layered closure of conjunctiva and Tenon’s capsule with Vicryl 7.0 and 10.0 Nylon sutures.

The XEN^®^ 63 µm (lumen diameter = 63 µm, length = 6 mm) implantation technique with the open conjunctiva approach is shown in Figure 1(A–I). Under sedation, retrobulbar anesthesia was performed by instilling 2 mL of a combination of 2% mepivacaine and 0.5% bupivacaine, except in eyes with severe glaucoma and a high risk of wipe-out. Initially, a superior temporal corneal 7.0 Vicryl traction suture was placed to optimize exposure of the conjunctiva. After an initial peritomy with Vanna’s scissors, sub-Tenon’s anesthesia (2% lidocaine combined with 2.5% epinephrine) was instilled. Subsequently, a superior fornix-based conjunctival flap was created, followed by a blunt dissection of Tenon’s capsule and conjunctiva with Westcott’s scissors. Subsequently, diathermy was applied to the blood vessels of the scleral bed to ensure appropriate hemostasis. A total of 0.02% mitomycin C (MMC) soaked up in Spongostan^TM^ (Johnson&Johnson, New Brunswick, NJ, USA) was applied for 2 min, followed by a gentle washing with balanced salt solution (BSS). The scleral tract was made using a bent 30G needle 2.0 mm away from the surgical limbus. The XEN implant was removed from its injector and manually positioned with atraumatic forceps through the scleral tract. When handling the device, caution was taken, avoiding its exposure to BSS to preserve its rigidity and to facilitate its insertion. In cases where the device had lost its rigidity and could not be introduced through the original scleral tract, a new scleral tract was performed using a 27G needle.

Adequate tubular filtration and the absence of peritubular filtration were assessed. A four-mirror gonioscopy lens was used during the surgical procedure to confirm the appropriate position of the device in the anterior chamber, ensuring a length of 3 mm under the conjunctiva, 2 mm in the intrascleral course, and 1 mm in the anterior chamber. (the 3-2-1 rule). If required, ocular tone was restored with BSS in the anterior chamber. At the surgeon’s discretion, Healaflow^®^ (MedicalMix, Spain), a filtration bleb modulator, was positioned on the scleral bed around the XEN implant as a space-occupying agent to separate the XEN lumen from the Tenon’s capsule. Finally, the Tenon’s capsule and conjunctiva were closed in planes, using Vicryl 7.0 and 10.0 Nylon sutures, respectively. Although Tenon’s capsule and conjunctiva may be sutured together, we believed that closing in planes avoids the posterior displacement of the Tenon’s capsule, preventing the development of avascular blebs. At the end of the procedure, intracameral 0.1 mL cefuroxime was instilled in the anterior chamber. 

### 2.4. Postoperative Regime, Procedures, and Care 

Patients had to discontinue the use of systemic and topical hypotensive medication at the time of surgery. Postoperative treatment included topical ciprofloxacin every 6 h for one week and topical phosphate dexamethasone every 2 h for three weeks with progressive tapering. Cycloplegic and atropine were used in cases of hypotony-associated complications such as hypothalamia, choroidal detachment, or hypotonic maculopathy. The frequency of the latter topical treatments was adjusted depending on the clinical condition and the ophthalmologist’s criteria.

Patients were evaluated postoperatively at the following time points: one day, one week, one month, two months, three months, six months, and one year after the surgery. The visiting scheme was modified depending on the postoperative evolution, the presence of complications, and the discretion of the surgeon. Secondary needling was performed in cases with bleb fibrosis or flat bleb with the presence of Tenon’s capsule obstructing the XEN distal portion, assessed by AS-OCT. The needling was performed with a 30 G hypodermic needle in the subconjunctival space, releasing the episcleral adhesions below and above the device. The use of MMC 0.01% was associated with the procedure at the ophthalmologist’s discretion. 

### 2.5. Surgical Success and Outcomes 

The primary outcome of this study was the number of cases achieving complete surgical success, defined as an IOP between 6 and 18 mmHg, with a reduction of ≥20% from preoperative values and no use of hypotensive medication. Moreover, qualified success included patients achieving an IOP of ≤18 mmHg and a ≥20% reduction from baseline while using glaucoma medications. Failure was defined as functional or structural POAG progression, IOP > 18 mmHg, loss of visual acuity secondary to hypotony-related complications or POAG progression 1 year after the surgery, the need for bleb surgical revision due to unmet IOP objective, or the need for additional glaucoma surgeries to achieve IOP control. The secondary outcomes of this study included the mean postoperative IOP and the mean number of required topical hypotensive medications 1 year after the procedure, BVCA (logMAR), MD of the visual field changes, needling requirements, intraoperative and postoperative complications, and the need for surgical revision or secondary procedures.

### 2.6. Statistical Analysis 

Statistical analyses were conducted with IBM SPSS version 30.0 (IBM Corp, United States). The normality of the data distribution was assessed with the Kolmogorov–Smirnov test. A paired T-Student test was used to assess statistical significance for mean value changes from baseline. In cases of variables with no normal distribution, the Wilcoxon test was performed. Kaplan–Meier survival analyses were used to evaluate both complete and qualified success rates. The prognostic factors for surgical failure were evaluated using Cox regression, including sex; age; POAG staging; preoperative lens status; number of preoperative glaucoma medications; previous SLT; preoperative BVCA (logMAR); preoperative visual field mean deviation; preoperative IOP; postoperative IOP at 1 day, 1 week, and 1 month; the use of bleb modulators; and the requirements of secondary needling in the first 3 months after the surgery. Variables with a *p* < 0.10 in univariate analysis were included in multivariate analysis. We performed multivariate analysis to adjust for potential cofounders and give robust statistical power to identify those variables associated with surgical failure. A *p* < 0.05 was considered statistically significant. The variance inflation factor was determined to assess multicollinearity between variables after the univariate analysis.

## 3. Results

### 3.1. Baseline Characteristics 

A total of 22 eyes of 20 patients were included in the study. The right eye was stented in 12 eyes. The mean age at the time of implantation was 70.1 ± 18.0 years. A total of 12 participants were male, and 10 were female. Two patients underwent bilateral surgery, and 9 eyes (40.9%) had undergone previous cataract surgery. Two cases (9.1%) had previous selective laser trabeculoplasty, and one eye (4.5%) had a previous history of LASIK for astigmatism and myopia. One eye (4.5%) underwent prior pars plana vitrectomy for retinal detachment three years prior to the XEN implantation. The preoperative mean baseline IOP was 21.9 ± 7.2 mmHg, the mean number of glaucoma medications was 2.4 ± 0.9, and the mean visual acuity (logMar) was 0.2 ± 0.4. Glaucoma staging was mild, moderate, and severe in eight, six, and eight eyes, respectively. The mean preoperative deviation was −6.36 ± 3.13 db. All patients underwent treatment with MMC 0.02% for 2 min. Sub-Tenon’s Healaflow^®^ was placed in 11 cases (50%). The demographic and baseline characteristics are summarized in Table 1.

### 3.2. Efficacy 

**Figure 2:**
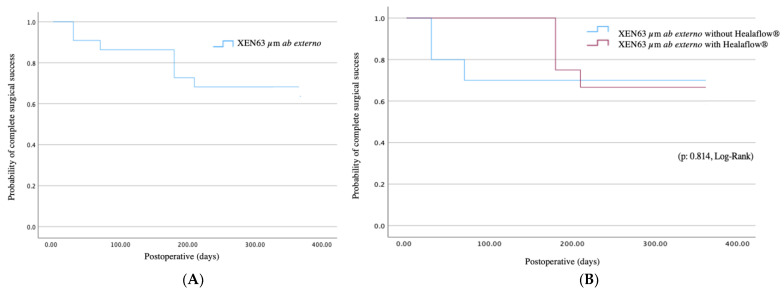
Cumulative probabilities of complete surgical success after XEN63 µm ab externo (**A**) and depending on the use of Healaflow (**B**).

Complete surgical success (IOP ≤ 18 mmHg, with a reduction of ≥20% from preoperative values and no use of hypotensive medication) was achieved in 14 cases (63.6%) (Figure 2A). Meanwhile, qualified success (IOP of ≤18 mmHg and a ≥20% reduction from baseline while using glaucoma medications) was achieved in 18 eyes (81.8%). No statistical differences were observed regarding the complete surgical success based on the use of Healaflow^®^ (Figure 2B).

Surgical failure occurred in four cases (18.2%) due to documented structural progression in three cases, and a surgical bleb revision in one case due to unmet target IOP. An additional surgical procedure was required in two patients to achieve IOP control. Univariate Cox regression demonstrated that secondary needling and an increased IOP 1 week and 1 month after surgery were associated with surgical failure. However, none of these variables showed significance in the multivariate Cox regression analysis (Table 2).

**Figure 3:**
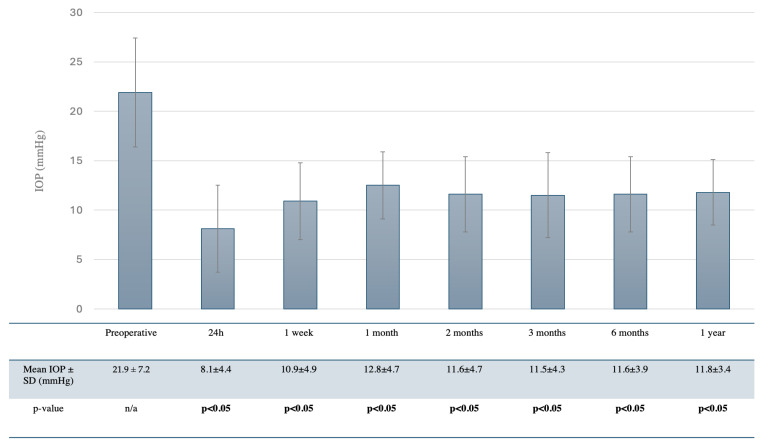
IOP measurements after ab externo open conjunctiva XEN implant. Bar diagram and table describing baseline and postoperative IOP values. Error bars indicated standard deviation of IOP.

One year after surgery, there was a statistically significant reduction in both the mean postoperative IOP (11.8 ± 3.4 mmHg, *p* < 0.05) and the mean number of hypotensive medications (0.2 ± 0.5, *p* < 0.05). Moreover, postoperative IOP was significantly lower than the preoperative values at all visits (*p* < 0.05). IOPs of 8.1 ± 4.4 mmHg, 10.9 ± 4.9 mmHg, 12.8± 4.7 mmHg, 11.6 ± 4.7 mmHg, 11.5 ± 4.3 mmHg, and 11.6 ± 3.9 mmHg were observed at 1 day, 1 week, 1 month, 2 months, 3 months, and 6 months, respectively (Figure 3). No statistical differences were observed concerning the BVCA (logMAR) and MD compared to preoperative data.

**Figure 4:**
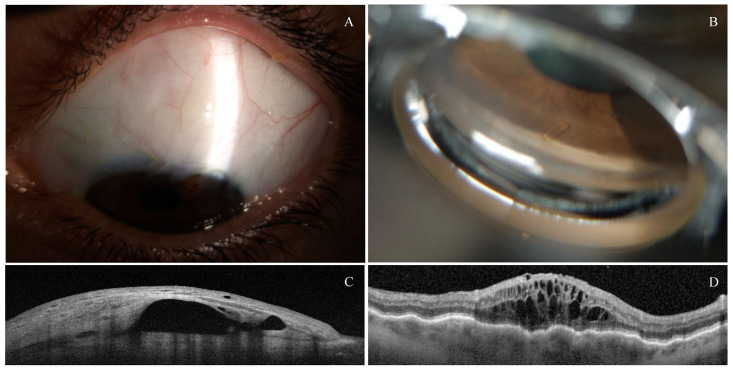
(**A**) Anterior segment photography of AEO XEN in LE, 6 months after surgery. (**B**) Implantation of the device through the scleral spur in the anterior chamber in LE through visualization with gonioscopy. (**C**) Filtration bleb with a hyporeflective area and subconjunctival cysts, depicted by AS-OCT. (**D**) Hypotonic maculopathy and cystoid diabetic macular edema, 1 week after XEN implantation, in a patient with moderate diabetic retinopathy.

Four eyes required secondary needling (18.2%), which all took place in the first two months after the initial surgery. Two of them had undergone Healaflow^®^ placement. As expected, there were no statistical differences in the needling requirements based on the presence of sub-Tenon’s Healaflow^®^. The correct position and filtration of the XEN device were assessed through gonioscopy and AS-OCT (Figure 4A–C).

### 3.3. Safety Profile 

**Figure 5:**
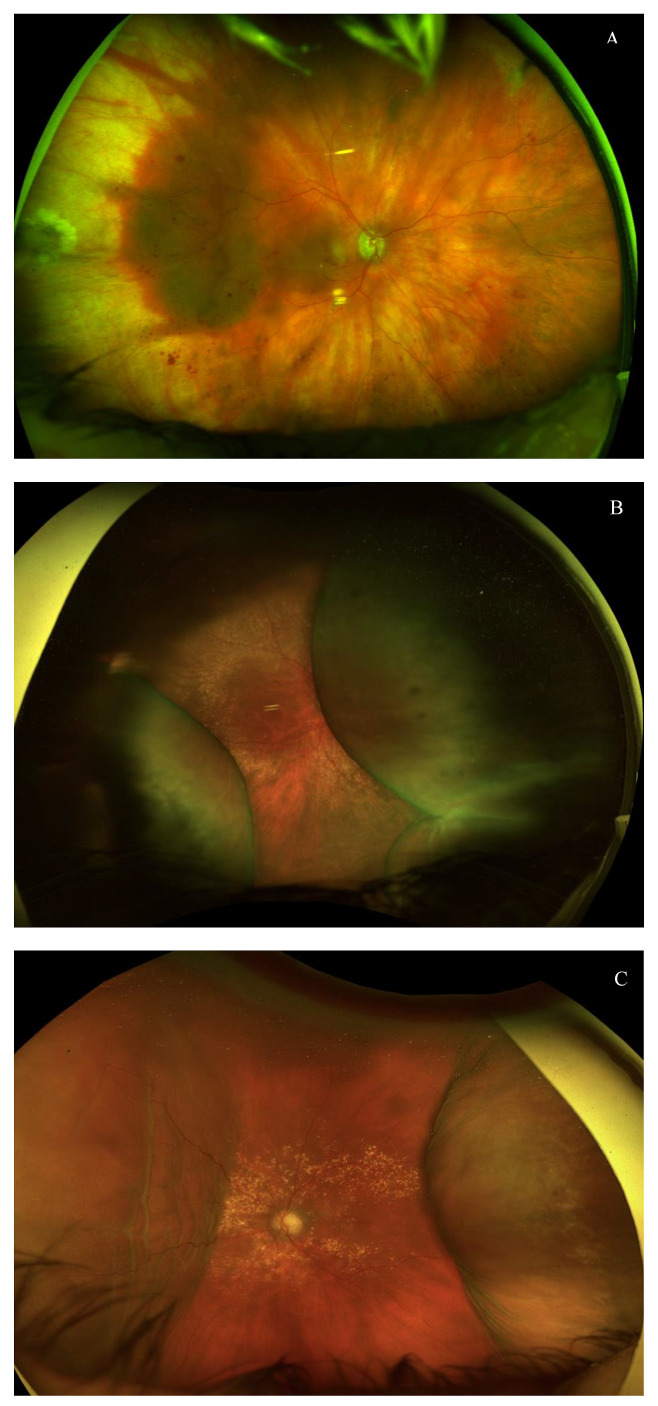
Hypotony-associated complications in three different eyes with AEO XEN^®^ 63 µm. (**A**) Localized hemorrhagic choroidal detachment 4 weeks after ab externo XEN63 µm implantation in a patient with moderate diabetic retinopathy. (**B**) Hypotony-associated multifocal choroidal serous detachment 3 weeks after ab externo XEN63 µm. (**C**) Resolving serous choroidal detachment with nasal choroidal folds after conservative management.

Intraoperative complications were infrequent and included two cases of self-limited hyphema. Seven eyes (31.8%) presented postoperative hypotony (defined as an IOP < 6 mmHg) 24 h after the surgery. All cases of hypotony resolved with topical treatment, with no persistent hypotony reported six months after the surgery. Hypotony-associated complications included one case of localized hemorrhagic choroidal detachment and four cases of serous choroidal detachment, one of them associated with hypotonic keratopathy and hypotonic maculopathy (Figure 4D and Figure 5A–C). Three patients with hypotony-related complications had an axial length > 25.0 mm. The hemorrhagic choroidal detachment occurred in an 88-year-old patient under acenocoumarol for atrial fibrillation with hypertension, type 2 diabetes mellitus and peripheral vasculopathy. All these complications occurred in the first month and evolved favorably with conservative management, rest, and adjusted topical treatment (topical mydriatics every 8 h and topical phosphate dexamethasone every 2 h with progressive tapering). No visual loss was associated in these cases, and there was no need for surgical revision. A case of a dysesthetic bleb with secondary Dellen required revision of the filtration bleb and placement of demarcation sutures, achieving a favorable postoperative outcome. Additionally, there was one case of postoperative cystoid macular edema (CME) that resolved with one dose of sub-Tenon’s triamcinolone.

## 4. Discussion

To the best of our knowledge, this is the first study evaluating the efficacy and safety of the ab externo open conjunctiva XEN 63 µm implant in POAG. Complete surgical success was obtained in 14 cases (63.6%) while qualified success was achieved in 18 eyes (82.8%). Moreover, we demonstrated that the standalone AEO XEN 63 µm implant significantly reduces mean IOP and the mean number of glaucoma medications 1 year after the procedure. In our study, IOP decreased from 21.9 ± 7.2 mmHg to 11.8 ± 3.4 mmHg, with a mean reduction of 10.2 ± 7.5 mmHg, 1 year after the surgery.

Ab interno standalone XEN^®^ 63 µm implant efficacy in POAG has been previously assessed. Thus, Fea et al. [15,17] demonstrated its short and long-term efficacy, standalone or combined with phacoemulsification, in POAG and pseudoexfoliative, uveitic, and traumatic glaucoma. They reported a comparable complete (60.9%) and qualified (73.9%) surgical success 18 months after the initial procedure [17]. Likewise, preoperative IOP was significantly lowered 18 months from 26.5 ± 8.2 mmHg to 14.2 ± 3.7 mmHg. Although the IOP reduction reported by Fea et al. was greater, this difference may be attributed to the higher observed baseline IOP [17]. Similarly, Martinez-de-la-Casa et al. [14] also reported promising and reliable data concerning AIC XEN63 µm in POAG. One year after the procedure, complete and qualified surgical success rates were 62.8% and 69.8%, respectively [14]. These results align with those obtained in our study. Concerning the reduction in the number of ocular hypotensive medications, the results obtained in our study are similar to those published elsewhere [14,15,17]. 

In our cohort, secondary needling was required in four cases (18.2%), and surgical revision was required in one case with a dysesthetic bleb. Compared to the ab interno approach, the incidence of secondary needling was higher than reported by Martinez-de-la-Casa et al. (5%) [14], inferior to Voykov et al. (33%) [18], and similar to the rate reported by Fea et al. (17.1%) [15]. It is important to underline that secondary needling was performed not based solely on IOP levels but also on the bleb morphology assessed at the slit lamp and the AS-OCT. This may justify the relatively high incidence of secondary needling in our cohort. In the AEO XEN 45, the reported incidence of secondary needling varies from 3.8% [11] to 26.7% [12]. It is unclear whether the AEO reduces the incidence of secondary needling compared to the AIC, as the studies conducted with the XEN45 offer mixed results [10,11]. 

As suggested by this article, the AEO XEN 63 µm may offer a safe alternative in the POAG treatment algorithm. Although rare, the intraoperative adverse events described include conjunctival buttonhole, XEN rupture, and vitreous prolapse in the anterior chamber [19,20]. In our cohort, ocular hypotony (IOP < 6 mmHg) was observed in seven cases (31.8%), predominantly in the first 24 h. Likewise, hypotony has been reported in up to 42.5% of patients undergoing AIC XEN 63 μm implant [14,15]. In our cohort, hypotony-related complications included four cases of serous choroidal detachment and one case of sectorial hemorrhagic choroidal detachment. All these complications manifested within the first postoperative month and showed improvement with topical treatment and conservative management, with no deterioration in visual acuity and no need for surgical revision. However, it is important not to underestimate these potential complications that may lead to irreversible visual loss. Thus, it is crucial to correctly individualize the surgical technique, considering the prior ophthalmological (myopia, aphakia) and systemic conditions (anticoagulant therapy and cardiovascular diseases) of the patients.

To our knowledge, there have been no studies conducted to evaluate the differences between AEO and AIC XEN^®^ 63 µm implant. However, there are studies comparing both surgical approaches for the 45 µm device. Most of these studies have shown a similar safety profile between both techniques for POAG. When evaluating the efficacy of both approaches, the results are more heterogeneous. While some authors, such as Ruda et al. [21], Yuan et al. [10], and Tan et al. [22], have shown comparable efficacy between both approaches, others, such as Hani El Helwe [11] et al., observed a higher surgical success rate with the AEO technique. 

Initially designed to be implanted through an ab interno approach, the ab externo XEN implantation has gained some popularity recently as it may offer additional advantages. Indeed, it allows for an extensive and thorough dissection of the conjunctiva and Tenon’s capsule, facilitating the sub-Tenon’s placement and localization and visualization of the device and the filtration bleb [23,24]. Likewise, it makes it possible to evaluate both tubular and peritubular filtration, ensuring the proper functioning of the implant and the absence of Tenon’s capsule entanglement at the lumen of the device. The open conjunctiva approach also minimizes manipulation of the anterior chamber and provides greater versatility, since it can be performed in any quadrant, which may be beneficial in cases with prior glaucoma surgeries. Additionally, an optimal application of MMC to the scleral bed is ensured, followed by extensive washout, reducing the risk of avascular bleb formation. The AEO technique allows for the placement of bleb spacers such as Healaflow^®^ or Ologen^®^ to improve the survival of the filtration bleb. Healaflow^®^, a cross-linked sodium hyaluronate, has demonstrated an adequate safety profile in glaucoma surgery, although without conclusive evidence of additional benefits [25,26,27]. In our study, the survival analysis did not show significant differences between the subgroups based on the use of Healaflow^®^. However, we observed a possible trend towards greater bleb survival in the short and medium term with its use. 

We acknowledge that the AEO approach may appear as a longer, more complex, and invasive procedure compared to the AIC technique. Likewise, the AEO technique deviates from the principles of traditional MIGS, whose main objective is to reduce intraocular pressure with an extremely high safety profile and a limited surgical manipulation at the expense of more limited efficacy. In contrast, we strongly believe that the AEO approach falls conceptually within the category of MIBS, a surgical approach that seeks a balance between minimal invasiveness and the generation of a functional bleb, with an efficacy closer to that of conventional filtering surgery, while maintaining an improved safety profile compared to it.

In the technique described in this article, a 30G needle is used to create the scleral tract instead of using the preloaded 27G injector. This contributes to limiting peritubular leakage, the main factor associated with early hypotony, guaranteeing the long-term functionality of the implant and reducing the incidence of secondary needling. The use of a 30G needle allows for an easier and more controlled approach when compared to the use of the injector. The latter, as it has a retractable system, requires an entrance closer to the limbus and has not been designed for an open conjunctiva approach. Although this technique implies a longer operating time, with the potential risk of device breakage, no intraoperative complications were reported. However, if the XEN has been hydrated, it may be difficult to implant the device through the scleral tract, and an alternative tract with a 27G needle can be created to implant the device. 

This study has several limitations. First, the small sample size and retrospective design may introduce biases that could affect the extrapolation of the results. Moreover, the filtration blebs were not evaluated by A-OCT in all cases, which makes it impossible to determine with certainty the existence of differences based on the use of Healaflow^®^. Although the surgical technique was standardized, the surgeries were performed by different surgeons, which could represent an additional bias. Likewise, short-term follow-up prevents evaluating the long-term efficacy of XEN. 

## 5. Conclusions

This study suggests that the AEO approach with a 30G needle for XEN63 µm implantation is an effective and safe surgical technique in the treatment of medically refractory POAG. Although hypotony is a frequent finding, most hypotony-related complications are self-limited and manageable with conservative treatment. Further studies are needed to determine the long-term efficacy of this technique.

## Figures and Tables

**Table 1:** Demographic and baseline characteristics.

Characteristics	Value (*n* = 22)
Follow-up time (days)	397 ± 25.4
Age, mean (y)	70.1 ± 18.0
Male sex	12 (54.5%)
Eye laterality (right)	12 (54.5%)
Preoperative mean BCVA (logMAR)	0.2 ± 0.4
Preoperative mean IOP (mmHg)	21.9 ± 7.2
Glaucoma staging *Mild* *Moderate* *Severe*	8 (36.4%) 6 (27.3%) 8 (36.4%)
Mean number of glaucoma medications	2.4 ± 0.9
Preoperative lens status (phakic)	13 (59.1%)
Mean visual field deviation (dB)	−6.36 ± 3.13
Previous selective laser trabeculoplasty (SLT)	2 (9.1%)
Previous ophthalmological procedures (time apart from XEN implant) *1 LASIK (15 years)* *1 Pars plana vitrectomy (3 years)* *9 phacoemulsification (all ≥ 6 months prior to XEN)*	1 (4.5%) 1 (4.5%) 9 (40.9%)

**Table 2:** Univariate and multivariate analysis of prognostic variables associated with surgical failure. Cox proportional hazards regression analyses were used for analyses; 1 week postoperative IOP was excluded as the variance inflation factor was > 10 when comparing 1 week and 1 month postoperative IOP. The multivariate model was adjusted for postoperative IOP at 1 month and secondary needling. HR = hazard ratio; CI = confidence interval; logMAR = logarithm of the minimum angle of resolution; IOP = intraocular pressure.

Variables	Univariate Analysis		Multivariate Analysis	
	**HR (95% CI)**	***p*-Value**	**HR (95% CI)**	***p*-Value**
Sex (male = 1)	0.764 (0.474–1.123)	0.435	-	-
Preoperative lens status (pseudophakic = 1)	0.921 (0.765–1.034)	0.765	-	-
Age (years)	1.025 (0.970–1.083)	0.376	-	-
Baseline VF MD (º)	1.112 (0.754–1.243)	0.567	-	-
Preoperative BVCA (logMAR)	0.984 (0.864–1.102)	0.430	-	-
Preoperative SLT	0.867 (0.645–1.429)	0.865	-	-
Number of preoperative medications	0.987 (0.433–2.249)	0.976	-	-
Use of Healaflow^®^	1.183 (0.281–4.972)	0.819	-	-
Preoperative IOP (mmHg)	0.840 (0.713–1.189)	0.336	-	-
24 h postoperative IOP (mmHg)	1.039 (0.888–1.217)	0.630	-	-
1 week postoperative IOP (mmHg)	**1.154 (1.029–1.294)**	**0.014**	-	-
1 month postoperative IOP (mmHg)	**1.137 (1.038–1.245)**	**0.005**	1.113 (0.986–1.256)	0.083
Secondary needling	**4.361 (1.502–9.936)**	**0.012**	2.753 (0.800–6.243)	0.538

## Data Availability

The datasets used and/or analyzed during the current study are available from the corresponding author upon reasonable request.

## Abbreviations

The following abbreviations are used in this manuscript:

AEO	Ab Externo With open conjunctiva
AIC	Ab Interno with closed conjunctiva
AS-OCT	Anterior Segment Optical Coherence Tomography
BSS	Balanced Salt Solution
BVCA	Best-Corrected Visual Acuity
CME	Cystoid Macular Edema
DB	Decibel
GATT	Gonioscopy-Assisted Transluminal Trabeculotomy
IOP	Intraocular Pressure
LASIK	Laser-Assisted In Situ Keratomileusis
logMAR	Logarithm of the Minimum Angle of Resolution
MD	Mean Deviation
MIBS	Minimally Invasive Bleb Surgery
MIGS	Minimally Invasive Glaucoma Surgery
MMC	Mitomycin C
NPDS	Non-Penetrating Deep Sclerectomy
OCT	Optical Coherence Tomography
ONH-OCT	Optical Coherence Tomography of the Optic Nerve Head
POAG	Primary Open-Angle Glaucoma
SLT	Selective Laser Trabeculoplasty
SPSS	Statistical Package for the Social Sciences
TBT	Trabeculectomy
VF	Visual Field

## References

[1] Spaeth G.L. (2021). European Glaucoma Society Terminology and Guidelines for Glaucoma, 5th Edition. Br. J. Ophthalmol..

[2] Vinod K., Gedde S.J., Feuer W.J., Panarelli J.F., Chang T.C., Chen P.P., Parrish R.K. (2017). Practice Preferences for Glaucoma Surgery. J. Glaucoma.

[3] Kirwan J.F., Lockwood A.J., Shah P., Macleod A., Broadway D.C., King A.J., McNaught A.I., Agrawal P. (2013). Trabeculectomy in the 21st Century: A Multicenter Study. Ophthalmology.

[4] Suñer I.J., Greenfield D.S., Miller M.P., Nicolela M.T., Palmberg P.F. (1997). Hypotony maculopathy after filtering surgery with mitomycin-C. Incidence and treatment. Ophthalmology.

[5] Lavia C., Dallorto L., Maule M., Ceccarelli M., Fea A.M. (2017). Minimally-Invasive Glaucoma Surgeries (MIGS) for Open Angle Glaucoma: A Systematic Review and Meta-Analysis. PLoS ONE.

[6] Bui T.T., Rosdahl J.A. (2022). Systematic Review of MIGS and Non-Penetrating Glaucoma Procedures for Uveitic Glaucoma. Semin. Ophthalmol..

[7] Traverso C.E., Carassa R.G., Fea A.M., Figus M., Astarita C., Piergentili B., Vera V., Gandolfi S. (2023). Effectiveness and Safety of Xen Gel Stent in Glaucoma Surgery: A Systematic Review of the Literature. J. Clin. Med..

[8] Balas M., Mathew D.J. (2023). Minimally Invasive Glaucoma Surgery: A Review of the Literature. Vision.

[9] Pirani V., Cavallero E., Cesari C., Virgili F., Ramovecchi V. (2024). *Ab Externo* Transconjunctival XEN® 45 Gel Stent Implantation: Efficacy and Safety of a New Surgical Technique. J. Curr. Glaucoma Pract..

[10] Yuan L., Rana H.S., Lee I., Lai G., Raiciulescu S., Kim W. (2023). Short-Term Outcomes of Xen-45 Gel Stent Ab Interno Versus Ab Externo Transconjunctival Approaches. J. Glaucoma.

[11] El Helwe H., Ingram Z., Neeson C.E., Falah H., Trzcinski J., Lin J.B., Solá-Del Valle D.A. (2024). Comparing Outcomes of 45 Xen Implantation Ab Interno with Closed Conjunctiva to Ab Externo with Open Conjunctiva Approaches. J. Glaucoma.

[12] Tan S.Y., Md Din N., Mohd Khialdin S., Wan Abdul Halim W.H., Tang S.F. (2021). Ab-Externo Implantation of XEN Gel Stent for Refractory Steroid-Induced Glaucoma After Lamellar Keratoplasty. Cureus.

[13] To L.K., Dhoot R.K., Chuang A.Z., Karimaghaei S., Guevara-Abadia F., Shah R.D., Feldman R.M. (2023). Defining the role of ab externo Xen gel stent in glaucomatous eyes with prior failed surgical intervention. Graefe’s Arch. Clin. Exp. Ophthalmol..

[14] Martínez-de-la-Casa J.M., Marcos-Parra M.T., Millá-Griñó E., Laborda T., Giménez-Gomez R., Larrosa J.M., Urcola A., Teus M.Á., Perucho-Martínez S. (2024). Effectiveness and safety of XEN63 in patients with primary-open-angle glaucoma. Sci. Rep..

[15] Fea A.M., Menchini M., Rossi A., Posarelli C., Malinverni L., Figus M. (2021). Early Experience with the New XEN63 Implant in Primary Open-Angle Glaucoma Patients: Clinical Outcomes. J. Clin. Med..

[16] Lavin-Dapena C., Cordero-Ros R., D’Anna O., Mogollón I. (2021). XEN 63 gel stent device in glaucoma surgery: A 5-years follow-up prospective study. Eur. J. Ophthalmol..

[17] Fea A.M., Menchini M., Rossi A., Posarelli C., Malinverni L., Figus M. (2022). Outcomes of XEN 63 Device at 18-Month Follow-Up in Glaucoma Patients: A Two-Center Retrospective Study. J. Clin. Med..

[18] Voykov B., Nasyrov E., Neubauer J., Gassel C.J. (2023). New XEN63 Gel Stent Implantation in Open-Angle Glaucoma: A Two-Year Follow-Up Pilot Study. Clin. Ophthalmol..

[19] Thatcher M.D., Coupal D.J., Cheng Y., Podbielski D.W. (2022). Short-Term Efficacy and Safety of Open Conjunctiva Ab Externo XEN45 Gel Stent Implantation in Glaucoma Patients. J. Glaucoma.

[20] Panarelli J.F., Yan D.B., Francis B., Craven E.R. (2020). XEN Gel Stent Open Conjunctiva Technique: A Practical Approach Paper. Adv. Ther..

[21] Ruda R.C., Yuan L., Lai G.M., Raiciulescu S., Kim W.I. (2023). Clinical Outcomes of Ab Interno Placement versus Ab Externo Placement of XEN45 Gel Stents. Ophthalmol. Glaucoma.

[22] Tan N.E., Tracer N., Terraciano A., Parikh H.A., Panarelli J.F., Radcliffe N.M. (2021). Comparison of Safety and Efficacy Between Ab Interno and Ab Externo Approaches to XEN Gel Stent Placement. Clin. Ophthalmol..

[23] Han K., Lee J., Moon S. (2023). One-Year Outcomes of Ab Externo XEN45 Gel Stent Implantation with an Open Conjunctiva Approach in Patients with Open-Angle Glaucoma. Korean J. Ophthalmol..

[24] Wy S., Shin Y.I., Kim Y.K., Jeoung J.W., Park K.H. (2023). Bleb Morphology on Anterior-Segment Optical Coherence Tomography after XEN Gel Stent Implantation. J. Clin. Med..

[25] Villarreal E., Berkowitz E., Tiosano B. (2023). XEN45 Gel Stent Combined with Healaflow Injectable Viscoelastic Implant. Case Rep. Ophthalmol. Med.

[26] Papaconstantinou D., Diagourtas A., Petrou P., Rouvas A., Vergados A., Koutsandrea C., Georgalas I. (2015). Trabeculectomy with Healaflow versus Trabeculectomy for the Treatment of Glaucoma: A Case-Control Study. J. Ophthalmol..

[27] Wu L., Liu J., Chang X., Zheng Y. (2021). The therapeutic effect of Healaflow in glaucoma surgery. Am. J. Transl. Res..

